# Supplementary material for: Monocyte-cancer cell fusion is mediated by phosphatidylserine—CD36 receptor interaction and induced by ionizing radiation
Source: PLoS One. 2025 Jan 3;20(1):e0311027. doi: 10.1371/journal.pone.0311027 (PMC11698428; doi:10.1371/journal.pone.0311027)
Supplement: Attachment — Submitted filename: PlosOne.pdf [file pone.0311027.s003.pdf]

Comments

In the present investigation “Monocyte-cancer cell fusion is mediated by phosphatidylserine - CD36 receptor interaction and induced by ionizing radiation”, Shabo et al have reported ionizing radiation resulted in the fusion of monocyte and tumor cells through the interaction between phosphatidylserine and CD36. The authors have co-cultured the tumor cell line and monocyte cell line and were subjected to ionizing radiation in the presence and absence of anti-CD36 antibodies to evaluate the interaction between phosphatidylserine and CD36 in the fusion of tumor cells and monocyte.

There are many studies studies regarding the fusion of cancer cells among themselves or/and with macrophages. However, to make this study more interesting and impactful, following comments can substantiate their findings.

1. The authors have presented and have explained the findings suitably. However, the language of the manuscript requires more improvement.
2. The authors have used the flow cytometry to show the fusion of the cells. However, the fusion of the cells should also be confirmed through microscopy. Since macrophages and monocytes are the scavenging and are involved in the phagocytosis, hence, microscopic images will confirm that the tumor cells are being phagocytosed not the apoptotic cell bodies.
3. The author should also measure the oxidative stress in the irradiated tumor and monocyte cell lines and fused tumor cells and cancer cell lines.
4. CD-36 have a prominent role in the scavenging potential of macrophages and phagocytosis. Therefore, the authors should also show the scavenging potential of the monocyte cell line in the presence of anti-CD36 antibodies.
5. The authors should confirm their findings with monocytes isolated from the human blood which will cement their findings.
6. The authors should show viability of the tumor cells and monocytes before and after the irradiation.
