# Supplementary material for: Monocyte-cancer cell fusion is mediated by phosphatidylserine—CD36 receptor interaction and induced by ionizing radiation
Source: PLoS One. 2025 Jan 3;20(1):e0311027. doi: 10.1371/journal.pone.0311027 (PMC11698428; doi:10.1371/journal.pone.0311027)
Supplement: Attachment — Submitted filename: Reviewer Comments_ PONE-D-23-37839_reviewer.doc [file pone.0311027.s004.doc]

**Reviewer Comments:**

The manuscript entitled " **Monocyte-cancer cell fusion is mediated by phosphatidylserine - CD36 receptor interaction and induced by ionizing radiation”** Current study highlights irradiation leads to a significant dose-dependent increase in the number of cancer cells expressing PS, while it does not affect the expression of CD36 in either THP-1 or MCF cells. Moreover, cell fusion generates new metastatic cancer cell/Tumor hybrid cells (THC) that exhibits the unique properties like abilities in colony formation, cell migration, invasion and over all acquired growth promoting properties. This is very an interesting study that can provide clue of targeted therapy for high grade tumor. However, I still have some queries and suggestion for the improvement of work qualities. Authors are advice to deal with the comments and adapt the manuscript accordingly, and submit a revised manuscript. At this stage manuscript does not hold merit of acceptance**.** I would like to review the manuscript again.

**Major concerns:**

1. As a results of Monocyte-cancer cell fusion a tumor hybrid cells (THC) get formed which exhibits a unique features like abilities of colony formation, cell migration, and invasion. In this study author has simply analyzed all data by FACS ARIA III. Since data is very unique and important so it should be validated by multiple parameters like morphological assessment by Immunofluorescence/ high contrast  laser scanning confocal microscopy ( LSCM ) images and some physiological assessment like colony formation assay, cell migration and invasion assay is very essential for further validation.
2. In the study, a single MCF7 luminal breast cancer cells have been selected for the study, which is more prone to form hybrids tumor cells than any other cells. Is there any specific reason of choosing this cell? Though cell fusion is a rare phenomenon, and the proportion of hybrid cells may be small compared to the total number of cells. Therefore it is advice to use other cell lines too for validation of the results along with normal cells.

**Manor concerns:**

1. **It is better to use term** tumor hybrid cells (THC) which is more specific and technical instead of **cancer cell fusion in running text.**
2. **Almost 80 to 90% references given to the article is quite old, kindly updates with some recent articles related to fields. There are some reference related to the field may be included i.e.** PMID: 30339548, PMID: 32612123, PMID: 28870843.
