# Supplementary material for: Monocyte-cancer cell fusion is mediated by phosphatidylserine—CD36 receptor interaction and induced by ionizing radiation
Source: PLoS One. 2025 Jan 3;20(1):e0311027. doi: 10.1371/journal.pone.0311027 (PMC11698428; doi:10.1371/journal.pone.0311027)
Supplement: Attachment — Submitted filename: Response to reviewers.docx [file pone.0311027.s005.docx]

Response to reviewers

**Point-by-point reply**

**Reviewer #1:** 
**1. The authors have presented and have explained the findings suitably. However, the language of the manuscript requires more improvement.**

Reply: We have reviewed and improved the language. Please see the text changes marked in yellow.

**2. The authors have used the flow cytometry to show the fusion of the cells. However, the fusion of the cells should also be confirmed through microscopy. Since macrophages and monocytes are the scavenging and are involved in the phagocytosis, hence, microscopic images will confirm that the tumor cells are being phagocytosed not the apoptotic cell bodies.**

Thank you for this important point of view. We attach a new figure with fluorescence microscopy staining CellTraicer Far red and Violet C. Cell sorting with FACS results in viable cells where tumor hybrids expressed both FR and V from CellTracer. In previous studies from our group, this protocol was used for cell hybridization/fusion, and viable tumor hybrid cells expressing phenotypic and genotypic traits from both parent cells were obtained. Please see these publications PMID: 28881654 and PMID: 26585897.

**3. The author should also measure the oxidative stress in the irradiated tumor and monocyte cell lines and fused tumor cells and cancer cell lines.**

Thanks for this interesting point. This study aims to show the interaction between CD36 and PS in relation to cell fusion rate. Quantifying oxidative stress in relation to fusion frequency and radiation dose is interesting, and we will explore it in future studies.

**4. CD-36 have a prominent role in the scavenging potential of macrophages and phagocytosis. Therefore, the authors should also show the scavenging potential of the monocyte cell line in the presence of anti-CD36 antibodies.**

This study investigates whether CD36 and PS are involved in fusion between a cancer cell and macrophages. Examining only scavenger function in monocytes is not expected to add information or affect the conclusions of this study.

**5. The authors should confirm their findings with monocytes isolated from the human blood which will cement their findings.**

Thanks for this valuable suggestion. In previous studies, we investigated the fusion between cancer cells and macrophages with monocytes from blood donors (PMID: 26585897 and PMID: 28881654). The origin of monocytes does not affect fusion frequency per se. This study aimed to highlight the outcome of the interaction between CD36 and PS in relation to cell fusion.

**6. The authors should show viability of the tumor cells and monocytes before and after the irradiation.**

Cell viability was estimated using trypan blue (Thermo Scientific, Waltham, USA). Data about cell viability are added to the manuscript text, and marked in yellow.

**Reviewer #2:**

1. **The investigation is exclusively conducted in vitro, posing a potential limitation in fully replicating the intricate dynamics of the in vivo microenvironment. Cellular interactions within the controlled laboratory conditions may diverge from those occurring within living organisms, possibly constraining the generalizability of the study's outcomes.**

Thank you for this important comment. Indeed, tumor microenvironment affects the biology of cancer cells. This study investigates how the interaction between CD36 and PS affects the fusion between macrophages and cancer cells. The study illustrates a specific cellular mechanism. Investigating this mechanism in the integrated context of the tumor microenvironment is interesting and will be the subject of future studies in our group.

2. **The utilization of specific cell lines (THP-1 and MCF-7) may not comprehensively represent the diverse array of cancer and immune cells present in authentic tumors. Different responses among various cancer types and subtypes may exist, and relying solely on a single breast cancer cell line might overlook this inherent variability.**

Thank you for this feedback. We agree that this mechanism might not occur in all solid tumors. As we suggested in previous comments, this study aims to illustrate the interaction between CD36 and PS in relation to cell fusion. We used MCF-7, a well-established cell line in which breast cancer tumor biology is investigated, and we used it in previous published studies.

3. **While the study underscores the significance of CD36 and phosphatidylserine in the fusion of leukocytes with cancer cells, it is imperative to acknowledge that additional molecular factors and signaling pathways could also play contributory roles in this intricate process. A more all-encompassing analysis of these involved molecules could yield a more comprehensive understanding.**

Cell fusion is a complicated multistep process that occurs between different types of cells, such as the fusion of sperm and egg and the fusion of myoblasts to form myotubes. Cell fusion involves the following steps: programming cells to make them competent for fusion, chemotaxis, recognition and attachment of the cells, cell membrane fusion, and finely generating a hybrid cell. Each fusion step is a complicated process, and exploring all steps in one study is impossible.

In the current study, we illustrate that the interaction of CD36 and PS is involved in the recognition process between MCF-7 cells and macrophages. We have added additional clarification on this topic in the Discussion. The text changes are marked in yellow.

4. **The primary focus of the study is on unraveling mechanisms at the cellular and molecular levels. Translating these discoveries into clinical applications necessitates further exploration to ascertain the clinical relevance and potential therapeutic implications of targeting CD36-phosphatidylserine interactions.**

Thank you for this essential point. This topic is briefly discussed in the section Discussion. It is a new topic, and knowledge of its clinical significance is limited. To avoid being too speculative, we have avoided developing a discussion on this theme more than it is described in the Discussion.

5. **Despite the compelling nature of the in vitro findings, the absence of in vivo validation imposes constraints on directly applying the results to the complexities of biological systems. Subsequent studies incorporating animal models or clinical samples hold promise for providing a more precise representation of the physiological relevance of these findings.**

Thanks for this feedback. In response, please see comments under point 1. Exploring this mechanism in vivo or organoid models is interesting, and we aim to investigate it in future studies.

6. **The study predominantly delves into the impact of ionizing radiation on leukocyte-cancer cell fusion. While this aspect is pivotal, an exclusive focus on a single treatment modality may fall short in capturing the diverse conditions encountered by cancer patients undergoing various therapeutic interventions.**

This study is a continuation of previous investigations where we studied the role of radiation in the occurrence of cell fusion and the development of radiation resistance in tumor hybrid cells (please see these publications PMID: 28881654 and PMID: 29725763). Therefore, we have limited this study to exploring cell fusion in relation to CD36 and PS interaction and radiation. Tentatively, chemotherapy treatment or exposure to inflammation might result in similar outcomes. Investigating all these factors becomes complicated as there are many types of cytostatic agents and causes of inflammation. Thus, it is difficult to limit these topics to the same study.

7. **The study overlooks the heterogeneity prevalent in cancer patients, encompassing variations in tumor microenvironments, patient responses to treatment, and the dynamic nature of cancer progression. Acknowledging and understanding these diverse factors is crucial for extending the applicability of the findings to a broader clinical context.**

Thanks for this interesting point. Cell fusion is a complicated process and constitutes several mechanisms leading to tumor heterogeneity. We agree with you, but unfortunately, this topic cannot be covered or investigated in this study.

**Reviewer #3:** 
**Major comments**

**1.As a results of Monocyte-cancer cell fusion a tumor hybrid cells (THC) get formed which exhibits a unique features like abilities of colony formation, cell migration, and invasion. In this study author has simply analyzed all data by FACS ARIA III. Since data is very unique and important so it should be validated by multiple parameters like morphological assessment by Immunofluorescence/ high contrast laser scanning confocal microscopy (LSCM ) images and some physiological assessment like colony formation assay, cell migration and invasion assay is very essential for further validation.**

Thanks for this interesting comment. We added fluorescence cell images as a new figure (Fig 2). Investigating the difference between tumor hybrid cells and their maternal cells is interesting but is not a goal of this study. We have ongoing projects with other questions to investigate morphological and functional differences between these cells.

**2. In the study, a single MCF7 luminal breast cancer cells have been selected for the study, which is more prone to form hybrids tumor cells than any other cells. Is there any specific reason of choosing this cell? Though cell fusion is a rare phenomenon, and the proportion of hybrid cells may be small compared to the total number of cells. Therefore, it is advice to use other cell lines too for validation of the results along with normal cells.**

This study continues previous investigations, in which we studied the role of radiation in cell fusion and the development of radiation resistance in tumor hybrid cells (please see these publications, PMID: 28881654 and PMID: 29725763). The idea of this study is to investigate a specific process (interaction between CD36 and PS). This process may occur in other tumor types and cell lines, but this topic is the subject of future studies where cell fusion and expression of PS are investigated in relation to other cancers.

**Manor concerns:**
**1. It is better to use term tumor hybrid cells (THC) which is more specific and technical instead of cancer cell fusion in running text.**

Thanks for the excellent point of view. According to your recommendation, we have changed the name of the hybrids to THC.

**2. Almost 80 to 90% references given to the article is quite old, kindly updates with some recent articles related to fields. There are some references related to the field may be included i.e. PMID: 30339548, PMID: 32612123, PMID: 28870843.**

Thanks for suggesting these studies. Following your recommendation, we have included the references PMID: 32612123 and PMID: 28870843 in the manuscript.
