# Supplementary material for: Monocyte-cancer cell fusion is mediated by phosphatidylserine—CD36 receptor interaction and induced by ionizing radiation
Source: PLoS One. 2025 Jan 3;20(1):e0311027. doi: 10.1371/journal.pone.0311027 (PMC11698428; doi:10.1371/journal.pone.0311027)
Supplement: Attachment — Submitted filename: Reviewer Comments_ PONE-D-23-37839_29042024.doc [file pone.0311027.s006.doc]

**Reviewer Comments:**

**The authors have addressed almost all the issues raised by the reviewer, but there is still some query:**

**Comments 1#** Authors has added fluorescence cell images in a new figure (Fig 2), which is simple MCF7, THP1 cells, and THP-1 cells stained with CD36 images using cellTraceTM staining. Authors should provide the cells images showing CD36, PS expression, and images of ionizing radiation showing their impact on CD36 and PS expression in cancer cells. In each case merge images should be provided. Further if possible try to capture the tumor hybrid cells images.
