# Supplementary material for: Monocyte-cancer cell fusion is mediated by phosphatidylserine—CD36 receptor interaction and induced by ionizing radiation
Source: PLoS One. 2025 Jan 3;20(1):e0311027. doi: 10.1371/journal.pone.0311027 (PMC11698428; doi:10.1371/journal.pone.0311027)
Supplement: Attachment — Submitted filename: R2 Response to Reviewer.doc [file pone.0311027.s007.doc]

**Response to Reviewer**

**Point-by-point reply**

**Reviewer #1:**

**The study's findings are very interesting; however, they lack a mechanistic approach. The authors must have included the experiments and findings with this work during revision rather than keeping them for a future goal. The addition of these experiments would have increased the impact of the present finding and made this work more interesting to the readers.**

**Reply:**

Thank you for this important viewpoint. As we stated in the previous revision, the aim of this study from the start was to explore whether CD36 and PS expression was involved in the cell fusion process and not to investigate its mechanistic interaction. We want to reassure you that our focus remains on this specific aspect of the cell fusion process, and we examine it in future studies. Moreover, we hope that the findings presented in this study will stimulate further research in other research groups.

**Reviewer #3:**

**The authors have addressed almost all the issues raised by the reviewer, but there is still some query:**

**Comments 1# Authors has added fluorescence cell images in a new figure (Fig 2), which is simple MCF7, THP1 cells, and THP-1 cells stained with CD36 images using cellTraceTM staining. Authors should provide the cells images showing CD36, PS expression, and images of ionizing radiation showing their impact on CD36 and PS expression in cancer cells. In each case merge images should be provided. Further if possible try to capture the tumor hybrid cells images.**

**Replay:**

Thank you for this question. The experiment ended a year ago, and unfortunately, we do not have MCF-7 THP-1 or hybrid cells left to take new images. During the planning of the study, we considered in our group that detection with FACS confirms the expression of these markers in respective cell lines. It would have been illustrative to have fluorescence images of the cells, but unfortunately, we do not have the opportunity to repeat the experiment.

**Reviewer #4: Minor point**

1. **Statistical analysis shown in figure 5, 6 and 7 needs to be elaborated by including the error bar, significance or level of significance etc. Also include the number of replicates used in experiment.**

**Reply:**

The bar charts are now converted to error bars. The experiments were repeated 3 times and we clarified this in M&M in the manuscript. These text changes are noted in yellow. The level of significance is indicated in the respective figure.

2. **Add the methodology of fluorescence microscope imaging in manuscript and include the magnification, scale bar etc. in figure 2.**

**Reply:**

Thank you for this remark. The method of fluorescence microscopy and image magnification is added to section M&M as a subhead “**Immunofluorescence microscopy”.** The text is marked in yellow.
